# Cardiac Fatigue in Male Athletes with Exercise-Induced Pulmonary Impairments After a Very Long-Distance Triathlon

**DOI:** 10.1007/s40279-024-02128-8

**Published:** 2024-10-16

**Authors:** Christophe Hédon, Fares Gouzi, Caroline Padovani, Iris Schuster, Claire Maufrais, Stéphane Cade, Frédéric Cransac, Gaspard Bui, Samuel Morcillo, Bronia Ayoub, Jérôme Thireau, Omar Izem, Cyril Reboul, Guillaume Walther, Maurice Hayot, Stéphane Nottin, Olivier Cazorla

**Affiliations:** 1 PhyMedExp, University of Montpellier, INSERM, CNRS, CHU Montpellier, 34295 Montpellier, France; 2 Cardiology Department, CHU Montpellier, Montpellier, France; 3 Physiology Department, CHU Montpellier, Montpellier, France; 4 CHU Nîmes, Nîmes, France; 5 University of Avignon, LaPEC UPR 4278, Avignon, France; 6 Cardiology Department, Clinique du Millénaire, Montpellier, France

## Abstract

**Introduction:**

Prolonged strenuous exercise can transiently decrease cardiac function. Other studies have identified three major exercise-induced pulmonary changes: bronchoconstriction, dynamic hyperinflation and pulmonary oedema with reduced alveolar–capillary membrane diffusing capacity. This study investigated whether athletes with one of these pulmonary dysfunctions following a very long-distance triathlon exhibit similar cardiac alterations as those without dysfunctions.

**Methods:**

Sixty trained male triathletes (age 39 ± 9 years) underwent baseline and post-race assessments, including echocardiography (with standard, 2D-strain and myocardial work assessments), spirometry and double-diffusion technique to evaluate alveolar–capillary membrane diffusing capacity for carbon monoxide (DM_CO_). Cardiac function in athletes with exercise-induced bronchoconstriction (> 10% decrease FEV_1_), dynamic hyperinflation (> 10% decrease inspiratory capacity) or impaired diffusion capacity (> 20% decrease DM_CO_/alveolar volume) were compared with those without these dysfunctions.

**Results:**

The race lasted 14 h 20 min ± 1 h 26 min. Both systolic and diastolic cardiac functions declined post-race. Post-race, 18% of athletes had bronchoconstriction, 58% dynamic hyperinflation and 40% impaired diffusing capacity. Right and left ventricular standard and 2D-strain parameters were similar before the race in all subgroups and changed similarly post-race, except E/E′, which decreased in the bronchoconstriction subgroup and increased in those with diffusion impairment. Global constructive work decreased by ~ 19% post-race (2302 ± 226 versus 1869 ± 328 mmHg%, *P* < 0.001), more pronounced in athletes with diffusion impairment compared with others (− 26 ± 13 versus − 15 ± 9%, *P* = 0.001) and positively correlated with DM_CO_/alveolar volume reduction.

**Conclusion:**

After a very long-distance triathlon, bronchoconstriction and hyperinflation were not associated with significant cardiac changes, whereas impaired alveolar–capillary membrane diffusing capacity was associated with a more significant decline in myocardial function. These findings highlight the complex relationship between pulmonary gas exchange abnormalities and cardiac fatigue following prolonged strenuous exercise.

**Supplementary Information:**

The online version contains supplementary material available at 10.1007/s40279-024-02128-8.

## Key Points

**Table:** 

After a very long-distance triathlon, athletes with exercise-induced bronchoconstriction or dynamic hyperinflation have similar cardiac fatigue, but athletes with alveolar–capillary membrane diffusing capacity impairment have a greater decrease in myocardial function. Prolonged strenuous exercise leads to an impairment of alveolar–capillary membrane diffusing capacity associated with myocardial fatigue.	

## Introduction

Prolonged strenuous exercise, such as a very long-distance triathlon, induces significant metabolic, muscular and cardiopulmonary stress [1]. Athletes, whether recreational or professional, may challenge their cardiac and pulmonary capacities to physiological extremes despite regular training.

Certain athletes may exhibit ‘cardiac fatigue’, characterized by a transient drop in both left and right ventricular systolic contractile function, along with impaired left ventricle diastolic filling, notably after long-distance triathlon [2–4]. While this post-exercise cardiac impairment is typically temporary, concerns arise regarding potential long-term deleterious adverse effects and the risk of ventricular arrhythmia with repeated participation in such events [5]. Cardiac function parameters are influenced by altered loading conditions at the race’s conclusion. However, employing new echocardiographic methods considering left ventricle pressure could reveal an intrinsic reduction in cardiac function [6].

At the pulmonary level, the high ventilatory demand during prolonged strenuous exercise induces mechanical stress on the pulmonary system and can lead to changes in ventilatory function [7], including changes in airway flow, such as exercise-induced bronchoconstriction [8], and in pulmonary mechanics, such as dynamic hyperinflation [9]. Moreover, studies observed mild alveolar–capillary oxygen diffusing capacity decrease in athletes after a marathon [10] or triathlon [11]. Those alterations could be linked to pulmonary interstitial oedema with increased radiologic lung density following these sporting events [12, 13]. However, the origin of this transient oedema, i.e. cardiac/haemodynamic origin or alveolar–capillary lesions, has yet to be clarified.

Cardiac and pulmonary systems are intimately linked through haemodynamic, mechanical and neurohumoral pathways. For example, airway obstruction (bronchoconstriction) and dynamic hyperinflation are usually associated with a substantial increase in end-expiratory pressure, which decreases the venous return, impairs ventricular loading conditions and could affect left ventricle ejection [14]. The reduced venous return could also influence pulmonary ventilation/perfusion mismatch, as shown in patients with heart failure with preserved ejection fraction [15]. Meanwhile, heart failure can lead to a decrease in airway flow due to peribronchovascular oedema [16] and a decrease in the oxygen diffusing capacity due to interstitial oedema, irrespective of the preservation or impairment of the left ventricle ejection fraction [17, 18]. Nevertheless, the association between cardiac and pulmonary function alterations observed in some athletes after prolonged strenuous exercise has never been investigated.

The aim of this study was to assess cardiac and pulmonary dysfunction immediately following a very long-distance triathlon. In a large cohort of athletes, we specifically assessed cardiac function, including left ventricle 2D-strain and myocardial work, and pulmonary function, including volume, airflow, the alveolar–capillary membrane diffusing capacity (DM) and pulmonary capillary blood volume (*V*_cap_). We investigated if athletes with pulmonary abnormalities, such as exercise-induced bronchoconstriction, dynamic hyperinflation or alveolar–capillary gas exchange anomalies, had more pronounced myocardial dysfunction than others. We hypothesized that, after the race, (1) myocardial function would be decreased, (2) pulmonary function would be altered in some athletes (evidence of exercise-induced bronchoconstriction, dynamic hyperinflation and DM reduction) and (3) the change in myocardial function would be more pronounced in case of pulmonary alterations.

## Methods

Additional information about methodologies is available in Supplementary Information.

### Study Population

This was a prospective study including 72 healthy male triathletes, aged 18–55 years, participating in a long-distance triathlon (EmbrunMan^®^: 3.8 km swim, 188 km cycling with a 4590 m positive elevation gain, and 42 km run). The non-inclusion criteria were subjects with known pulmonary or heart disease, rhythm disorder and presence of at least one cardiovascular risk factor. This study was approved by the French institutional review board CPP SUD MEDITERRANEE I on 11 April 2018. All patients enrolled in the study provided written consent.

A medical evaluation of all participants was performed at rest 24–72 h before the race and after the race. The medical evaluation included vital signs (heart rate, electrocardiogram) and basic anthropometry (stature and mass). Next, participants completed echocardiography with a concomitant systolic and diastolic blood pressure measure (52 ± 17 min after crossing the finish line). Lastly, pulmonary function tests including spirometry and resting lung diffusing capacity were assessed (84 ± 24 min after crossing the finish line).

### Echocardiography

Echocardiographic images were obtained using five commercially available ultrasound systems (Vivid IQ, Vivid S70 and Vivid E95, 3Sc-RS probe, General Electric). Images were obtained by experienced sonographers followed a standardized protocol. Images, recorded with five electrocardiogram (ECG)-triggered cardiac cycles, were analysed offline using EchoPac 203 software (General Electric), averaging data from three cardiac cycles to assess standard and 2D-strain echocardiographic parameters as previously [19] (see supplemental methods). Myocardial work involved integrating the left ventricle global longitudinal strain and intra-left ventricle pressure, estimated non-invasively from brachial systemic blood pressure measurements, taken just before echocardiography, as described by Russel et al. [6]. A left ventricle pressure–strain loop curve was constructed, and additional parameters were calculated, including the global work index measured as the total work from mitral valve closure to opening, representing the pressure–strain loop area. Global constructive work was defined as myocardial work during segmental shortening in systole, and segmental lengthening during the isovolumetric relaxation phase. Global wasted work was the work performed during lengthening in systole and shortening in isovolumic relaxation associated with energy loss. Global work efficiency was expressed as the ratio between myocardial global constructive work and the sum of global constructive work and global wasted work.

### Pulmonary Function Testing

Spirometry was conducted using a single spirometer (Medisoft-MGCd, Sorinnes, Belgium) in accordance with international recommendations [20]. A consistent operator performed slow vital capacity followed by forced expiration manoeuvres, each repeated at least three times for each athlete. The measured spirometry parameters included forced expiratory volume in one second (FEV_1_), slow vital capacity, inspiratory capacity and inspiratory reserve volume. Exercise-induced bronchoconstriction was defined as a post-race drop of more than 10% in FEV_1_ compared with pre-race, following established guidelines [21]. Dynamic hyperinflation of the lung was identified by a 10% decrease in the post-race inspiratory capacity, a threshold considered clinically significant [22]. The alveolar–capillary diffusing capacity was assessed as previously described [23] by studying the lung diffusing capacity for carbon monoxide (DL_CO_) and nitric oxide (DL_NO_) using the Hyp’Air system (Medisoft-MGCd, Sorinnes, Belgium). Measurements for DL_CO_ and DL_NO_ were conducted simultaneously in duplicate, with a 4-min rest between each measurement, according to the ERS standardization for single-breath determination of nitric oxide uptake in the lung [24]. Alveolar volume during breath hold was calculated using the He-dilution technique. The alveolar–capillary membrane diffusing capacity for carbon monoxide (DM_CO_) and pulmonary capillary blood volume (*V*_cap_) were calculated using the Roughton and Forster method [25]. We defined a significant impairment in alveolar–capillary membrane diffusing capacity as a post-race decrease in DM_CO_ per unit effective alveolar volume greater than 20%, compared with pre-race. This threshold is considered clinically relevant, as it approximates the difference observed between heart failure patients and healthy subjects [17]. Furthermore, in healthy individuals, meaningful changes in DM_CO_ should exceed the reported 12% spontaneous variability in DM_CO_ measurements [26]. Pulsed arterial oxygen saturation (SpO2) was measured using a Masimo SET^®^ Rad-5 pulse oximeter with a finger sensor (Masimo, Danderyd, Sweden). To assess whether the changes in pulmonary function could be associated with changes in cardiac function, we divided the athletes into two subgroups according to the presence or not of either (1) exercise-induced bronchoconstriction, (2) dynamic hyperinflation or (3) alveolar–capillary membrane diffusing capacity impairment.

### Statistical Analysis

Quantitative variables are presented as mean ± standard deviation (SD). Comparisons between the pre-race and post-race measurements were done using a paired Student’s* t*-test, after checking the normal data distribution. Subgroup comparisons whether an athlete had pulmonary impairment or not were done using unpaired Student’s *t*-tests. Correlations between athletes’ general characteristics and cardiac and/or pulmonary modifications were assessed using the Pearson correlation test. All *P* values were adjusted on the basis of the false discovery rate (FDR) for multiple tests applied to all *P* values together. Significance was set at 0.05 for all comparisons. The analyses were performed using GraphPad Prism software (version 8.2.1).

## Results

### Study Population

**Fig. 1:**
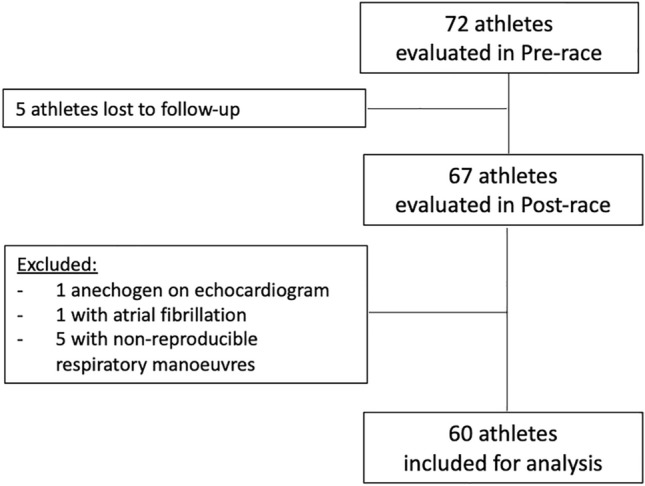
Flow chart of enrolled athletes

Sixty of the 72 athletes examined pre-race were included in the final analysis (Fig. 1). The participants were on average 39 ± 9 years old and had trained 12 ± 3 h/week in the few months prior to the triathlon. Thirty-seven athletes (62%) had participated at least once in a very long-distance triathlon. The average race time was 14 h 20 min ± 1 h 26 min (range 11 h 30 min to 17 h 10 min).

### Cardiac Function in the Overall Population

**Fig. 2:**
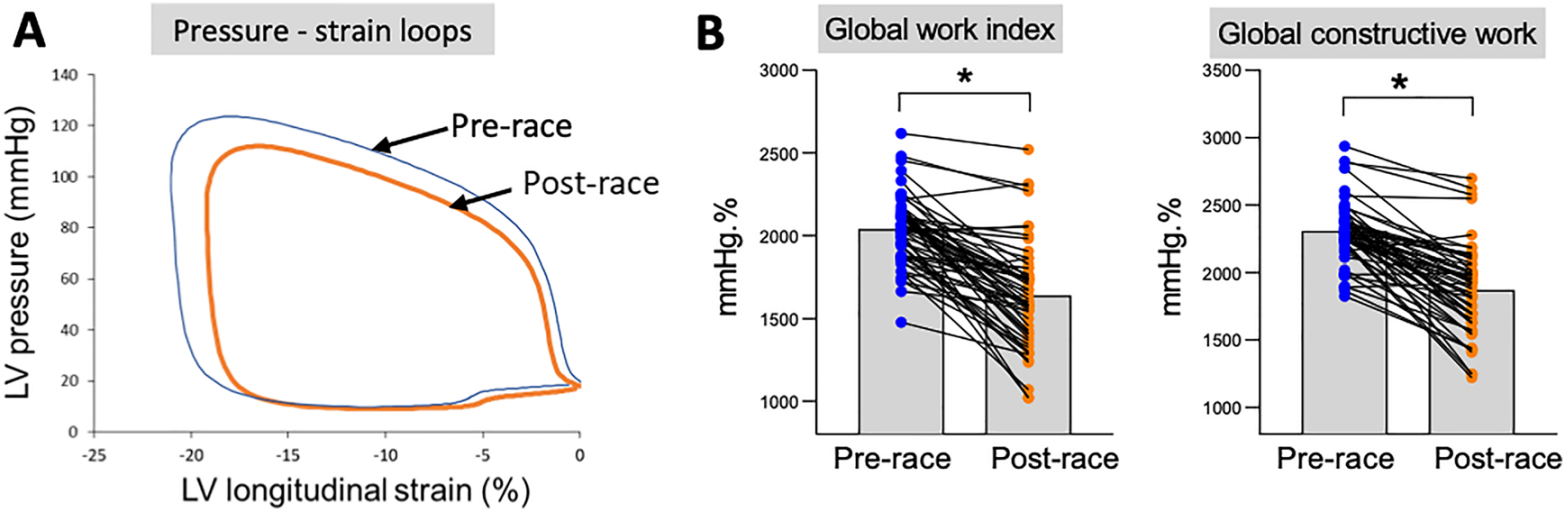
Myocardial work before and after a very long-distance triathlon. **A** Non-invasive left ventricular pressure–strain loops averaged from the whole population were decreased post-race. **B** Individual (points) and mean (box plots) of myocardial work index pre-race and post-race. The global work index (area under the curve of pressure–strain loops) decreased post-race. Global constructive work, which is the work during segmental shortening in systole, and segmental lengthening during the isovolumetric relaxation phase decreased post-race. *Significance *P* < 0.05 (paired *t*-test, adjusted on the basis of FDR) (*N* = 60)

Cardiac data pre-race and post-race and the percentage change are presented in Table 1. All the athletes had a normal cardiac morphology and function pre-race. After the race, left ventricle ejection fraction, global longitudinal strain and stroke volume had decreased while the heart rate increased, resulting in unchanged cardiac output. We enhanced the myocardial function assessment by measuring the left ventricle myocardial work, which accounted for changes in loading conditions. Indeed, diastolic and systolic blood pressure both decreased after the race. From the pressure–strain loop constructed, the global work index was calculated as the total work performed by the left ventricle. The global work index declined by 20% owing to a decrease in both left ventricle global longitudinal strain and intra-left ventricle pressure, as shown by the averaged pressure–longitudinal strain loops (Fig. 2A, B). The global work index includes global constructive work, defined as myocardial work during segmental shortening in systole, segmental lengthening during the isovolumetric relaxation phase and global wasted work associated with energy loss. Global constructive work had significantly decreased by 19% while global wasted work increased post-race in comparison with the pre-race findings (Fig. 2B). Global work efficiency, constructive work divided by the sum of constructive work and wasted work, hence had decreased. The end-diastolic volume and diastolic function indexes including peak E and peak E′ had also decreased. The right ventricle was altered with increased end-diastolic and end-systolic areas and decreased systolic function with a reduction in fractional area shortening, tricuspid annular plane systolic excursion (TAPSE), right ventricle peak S′ velocity and longitudinal strain.

**Table 1:** Left and right ventricular parameters before (pre-race) and after (post-race) the very long-distance triathlon

	Pre-race	Post-race	% change	Adjusted *P* value
Heart rate (bpm)	59 ± 9	70 ± 9	21 ± 18	** < 0.001**
Systolic blood pressure (mmHg)	124 ± 8	112 ± 11	− 10 ± 8	** < 0.001**
Diastolic blood pressure (mmHg)	67 ± 7	65 ± 8	− 3 ± 13	**0.032**
*LV morphology and systolic function*				
LV end-diastolic volume (mL)	160 ± 25	149 ± 22	− 6 ± 11	** < 0.001**
LV end-systolic volume (mL)	52 ± 12	53 ± 11	5 ± 22	0.434
LV ejection fraction (%)	68 ± 5	65 ± 5	− 5 ± 9	** < 0.001**
Stroke volume (mL)	101 ± 21	87 ± 18	− 12 ± 19	** < 0.001**
Cardiac output (L min^−1^)	5.9 ± 1.3	6.2 ± 1.2	8 ± 26	0.146
LV global longitudinal strain (%)	− 21.3 ± 1.9	− 19.6 ± 2.0	− 8 ± 10	** < 0.001**
LV basal circumferential strain (%)	− 18.2 ± 3.5	16.0 ± 2.8	− 9 ± 24	** < 0.001**
LV apical circumferential strain (%)	− 23.0 ± 3.8	− 19.6 ± 4.1	− 12 ± 25	** < 0.001**
*LV myocardial work*				
Global work index (mmHg%)	2037 ± 211	1637 ± 316	− 20 ± 14	** < 0.001**
Global constructive work (mmHg%)	2302 ± 226	1869 ± 328	− 19 ± 12	** < 0.001**
Global wasted work (mmHg%)	83 ± 39	104 ± 46	49 ± 107	**0.007**
Global work efficiency (%)	95.6 ± 1.7	94.0 ± 2.4	− 2 ± 3	** < 0.001**
*LV diastolic function*				
Peak E (cm s^−1^)	78 ± 14	62 ± 12	− 20 ± 15	** < 0.001**
Peak A (cm s^−1^)	54 ± 13	49 ± 9	− 5 ± 22	**0.004**
E/A	1.5 ± 0.5	1.3 ± 0.3	− 12 ± 18	** < 0.001**
Peak E′_lat_ (cm s^−1^)	− 13.0 ± 2.4	− 11.1 ± 2.5	− 13 ± 22	** < 0.001**
E/E′_lat_	6.2 ± 1.6	5.8 ± 1.2	− 3 ± 29	0.051
*RV morphology and systolic function*				
RV end-diastolic area (cm^2^)	26.1 ± 4.7	27.8 ± 4.1	8 ± 16	**0.002**
RV end-systolic area (cm^2^)	14.4 ± 3.3	16.5 ± 3.6	17 ± 21	** < 0.001**
RV fractional area shortening (%)	44.7 ± 9.3	40.5 ± 9.5	− 7 ± 24	**0.002**
TAPSE (mm)	27.7 ± 4.4	24.6 ± 3.5	− 10 ± 12	** < 0.001**
RV peak S′ (cm s^−1^)	11.8 ± 1.8	10.5 ± 2.0	− 11 ± 17	** < 0.001**
RV longitudinal strain (%)	− 26.7 ± 4.0	− 23.9 ± 4.8	− 9 ± 23	** < 0.001**

Values are presented as mean ± SD

LV, left ventricular; RV, right ventricular; TAPSE, tricuspid annular plane systolic excursion

*P* values in bold means statistical difference *P* < 0.05  assessed by paired *t*-test adjusted on the basis of FDR (*n* = 60)

### Pulmonary Function in the Overall Population

All pulmonary data pre-race and post-race and the percentage changes are presented in Table 2. All athletes exhibited normal airflow and lung volume pre-race, whereas FEV_1_ and the vital capacity had significantly decreased post-race. Eleven athletes (i.e. 18% of the total population) presented with exercise-induced bronchoconstriction. The inspiratory capacity had also dropped in the whole population. Dynamic hyperinflation was observed in 35 athletes (i.e. 58% of the total population). Alveolar–capillary gas exchange had also significantly altered post-race. The lung diffusing capacity for NO (DL_NO_) decreased to a greater extent compared with lung diffusing capacity for CO (DL_CO_), leading to a decreased DL_NO_/DL_CO_ ratio post-race. There was, thus, a decrease in the DM_CO_/*V*_cap_ ratio post-race. The calculations revealed that the alveolar volume-adjusted DM_CO_ had decreased to a greater extent than the alveolar volume-adjusted *V*_cap_, in line with the alveolar–capillary membrane conductance impairment indexed by the decrease in their DM_CO_ per unit effective alveolar volume. Twenty-four athletes (40%) experienced significative impaired alveolar–capillary membrane diffusing capacity (DM_CO_ per unit effective alveolar volume greater than 20%). The pulsed arterial oxygen saturation was not modified post-race in the whole population.

**Table 2:** Functional pulmonary parameters obtained before (pre-race) and after (post-race) the very long-distance triathlon

	Pre-race	Post-race	% change	Adjusted *P* value
SpO_2_ (%)	97.4 ± 1.3	97.0 ± 1.7	0 ± 2	0.228
*Pulmonary volumes and flows*				
FEV_1_ (L)	4.6 ± 0.5	4.4 ± 0.6	− 4 ± 10	**0.003**
Vital capacity (L)	5.9 ± 0.7	5.4 ± 0.7	− 8 ± 8	** < 0.001**
Inspiratory capacity (L)	3.9 ± 0.5	3.4 ± 0.5	− 13 ± 13	** < 0.001**
Inspiratory reserve volume (L)	2.5 ± 0.6	2.1 ± 0.6	− 18 ± 23	** < 0.001**
*Alveolar–capillary diffusion*				
DL_CO_ (mL min^−1^ mmHg^−1^)	40.2 ± 5.2	35.8 ± 4.4	− 10 ± 9	** < 0.001**
DL_NO_ (mL min^−1^ mmHg^−1^)	219 ± 40	187 ± 25	− 14 ± 8	** < 0.001**
K_CO_ (mL min^−1^ mmHg^−1^ L^−1^)	5.2 ± 0.7	4.9 ± 0.6	− 5 ± 7	** < 0.001**
DL_NO_/DL_CO_	5.5 ± 0.5	5.2 ± 0.3	− 4 ± 8	** < 0.001**
*V*_cap_ (mL)	84 ± 13	77 ± 10	− 7 ± 11	** < 0.001**
*V*_cap_/alveolar volume (mL/L)	10.9 ± 2.0	10.5 ± 1.5	− 3 ± 10	**0.028**
DM_CO_ (mL min^−1^ mmHg^−1^)	281 ± 86	213 ± 46	− 21 ± 18	** < 0.001**
DM_CO_/alveolar volume (mL min^−1^ mmHg L^−1^)	36.2 ± 10.0	28.8 ± 5.3	− 17 ± 18	** < 0.001**
DM_CO_/*V*_cap_ (min^−1.^mmHg^−1^)	3.5 ± 1.3	2.8 ± 0.6	− 12 ± 27	** < 0.001**

Values are presented as mean ± SD

DL_CO_, lung diffusing capacity for carbon monoxide; DL_NO_, lung diffusing capacity for nitric oxide; DM_CO_, alveolar–capillary membrane diffusing capacity for carbon monoxide; FEV_1_, forced expiratory volume in 1 s; SpO_2_, pulsed arterial oxygen saturation; *V*_cap_, pulmonary capillary blood volume

*P* values in bold means statistical difference *P* < 0.05  assessed by paired *t*-test adjusted on the basis of FDR (*n* = 60)

### Cardiac Function in Athletes with Exercise-Induced Bronchoconstriction, Hyperinflation, Diffusion Impairment

The general characteristics and the change in the pulmonary and cardiac function parameters of the different pulmonary alteration subgroups are presented in Table 3. The time to conduct the post-race pulmonary assessment did not differ between subgroups. All subgroups exhibited similar initial cardiac or pulmonary functions at pre-race, except for global work index, which was higher pre-race in athletes with exercise-induced bronchoconstriction compared with those without bronchoconstriction (*P* = 0.02, Supplemental Table S1).

**Table 3:** Athlete characteristics and change in the pulmonary and cardiac function parameters in different subgroups

	Bronchoconstriction		Hyperinflation		Diffusion impairment	
	No (*n* = 49)	Yes (*n* = 11)	No (*n* = 25)	Yes (*n* = 35)	No (*n* = 36)	Yes (*n* = 24)
*Pulmonary function*						
%Δ SpO_2_	− 0.2 ± 1.7	0.7 ± 2.8	− 0.6 ± 2.4	− 0.1 ± 1.5	− 0.2 ± 2.1	− 0.5 ± 1.7
%Δ FEV_1_	** − 0.6 ± 6.0**	− 18.9 ± 10.1*	− 1.3 ± 6.5	− 6.5 ± 11.8	− 4.2 ± 11.9	− 3.7 ± 6.7
%Δ inspiratory capacity	** − 11.1 ± 12.4**	− 19.7 ± 14.4*	** − 0.8 ± 8.5**	− 21.2 ± 8.3*	− 11.1 ± 14.2	15.8 ± 10.8
%Δ DM_CO_/alveolar volume	− 16.6 ± 18.9	− 17.5 ± 16.7	− 9.4 ± 6.8	− 9.6 ± 7.1	** − 5.3 ± 11.0**	− 34.7 ± 12.3*
*LV function*						
%Δ LV end-diastolic volume	− 5.7 ± 11.6	− 8.3 ± 8.1	− 5.4 ± 11.3	− 6.8 ± 10.9	− 6.7 ± 12.3	− 5.1 ± 9.1
%Δ LV ejection fraction	− 4.7 ± 8.4	− 3.6 ± 11.6	− 6.7 ± 8.7	− 2.9 ± 8.9	− 3.3 ± 8.5	6.2 ± 9.6
%Δ cardiac output	5.4 ± 24.8	21.7 ± 29.7	12.9 ± 27.1	5.4 ± 25.7	3.6 ± 22.5	15.4 ± 30.8
%Δ E/E′_lat_	**0.4 ± 29.3**	− 20.1 ± 22.3*	− 0.5 ± 37.2	− 5.4 ± 21.9	** − 10.0 ± 22.0**	8.0 ± 35.6*
%Δ longitudinal strain	− 10.9 ± 9.4	− 5.9 ± 12.4	− 11.0 ± 9.0	− 8.9 ± 10.8	− 9.6 ± 10.2	− 9.9 ± 10.4
%Δ basal circumferential strain	− 11.7 ± 27.3	− 14.7 ± 15.6	− 10.4 ± 25.2	− 8.4 ± 24.0	− 8.2 ± 26.4	− 10.5 ± 21.1
%Δ apical circumferential strain	− 12.4 ± 25.2	− 6.6 ± 20.6	− 6.3 ± 29.6	− 14.3 ± 20.5	− 11.7 ± 22.7	− 11.1 ± 28.2
%Δ global work index	− 19.0 ± 13.0	− 16.7 ± 14.8	− 17.7 ± 11.5	− 21.0 ± 14.9	** − 14.7 ± 11.7**	− 27.4 ± 13.2*
%Δ global constructive work	− 18.5 ± 11.5	− 16.7 ± 12.5	− 19.3 ± 10.5	− 18.9 ± 12.7	** − 14.6 ± 8.7**	− 25.9 ± 13.1*
*RV function*						
%Δ RV end-diastolic area	8.4 ± 16.3	6.6 ± 15.6	10.8 ± 17.0	6.2 ± 15.3	5.7 ± 13.7	12.1 ± 19.0
%Δ TAPSE	− 10.8 ± 11.8	− 5.1 ± 11.1	− 9.6 ± 10.1	− 9.8 ± 12.8	− 11.2 ± 11.6	− 7.6 ± 12.2
%Δ RV peak S’	− 12.0 ± 15.9	− 4.0 ± 20.8	− 9.0 ± 16.4	− 11.7 ± 17.5	− 8.1 ± 18.0	− 15.7 ± 14.0
%Δ RV longitudinal strain	− 9.8 ± 25.0	− 6.5 ± 21.0	− 7.2 ± 32.6	− 10.3 ± 13.8	− 7.7 ± 28.3	− 10.9 ± 12.5
*General characteristics*						
Age	39 ± 9	38 ± 9	**42 ± 8**	37 ± 9*	40 ± 9	38 ± 8
BMI	22.6 ± 1.8	23.6 ± 2.3	22.8 ± 2.2	22.8 ± 1.8	22.9 ± 2.1	22.6 ± 1.8
Years of triathlon experience	7 ± 5	10 ± 8	8 ± 6	7 ± 6	8 ± 7	7 ± 5
*N* of ultratriathlon in the past	1.2 ± 1.6	1.5 ± 2.3	0.9 ± 1.1	1.5 ± 2.0	1.2 ± 1.6	1.1 ± 1.6
Training load per week (h)	12 ± 3	12 ± 3	12 ± 3	13 ± 3	12 ± 3	12 ± 4
Total race time (h:min)	14 h 05 ± 1 h 22	14 h 23 ± 1 h 28	14 h 39 ± 1 h 09	14 h 06 ± 1 h 36	**14 h 40 ± 1 h 23**	13 h 42 ± 1 h 21*
Swimming time (h:min)	1 h 11 ± 0 h 10	1 h 17 ± 0 h 09	1 h 16 ± 0 h 08	1 h 15 ± 0 h 10	1 h 16 ± 0 h 10	1 h 14 ± 0 h 07
Cycling time (h:min)	8 h 08 ± 0 h 56	8 h 10 ± 0 h 44	8 h 22 ± 0 h 44	8 h 01 ± 0 h 47	8 h 12 ± 0 h 43	8 h 06 ± 0 h 51
Running time (h:min)	4 h 43 ± 0 h 39	4 h 43 ± 0 h 42	4 h 50 ± 0 h 35	4 h 38 ± 0 h 45	**4 h 56 ± 0 h 39**	4 h 23 ± 0 h 31*

Subgroups were clustered according to the presence or not of exercise-induced bronchoconstriction, dynamic hyperinflation and alveolar–capillary membrane diffusing capacity impairment after the race

Values are presented as mean ± SD

%Δ, % of change between post-race and pre-race; BMI; body mass index; DM_CO_, alveolar–capillary membrane diffusing capacity for carbon monoxide; FEV_1,_ forced expiratory volume in 1 s; LV, left ventricular; RV, right ventricular; SpO_2_, pulsed arterial oxygen saturation; TAPSE, tricuspid annular plane systolic excursion

*P* values in bold means and with Asterisk means statistical difference *P* < 0.05 assessed by unpaired* t*-test, adjusted on the basis of FDR between the conditions yes and no (*n* = 60)

**Fig. 3:**
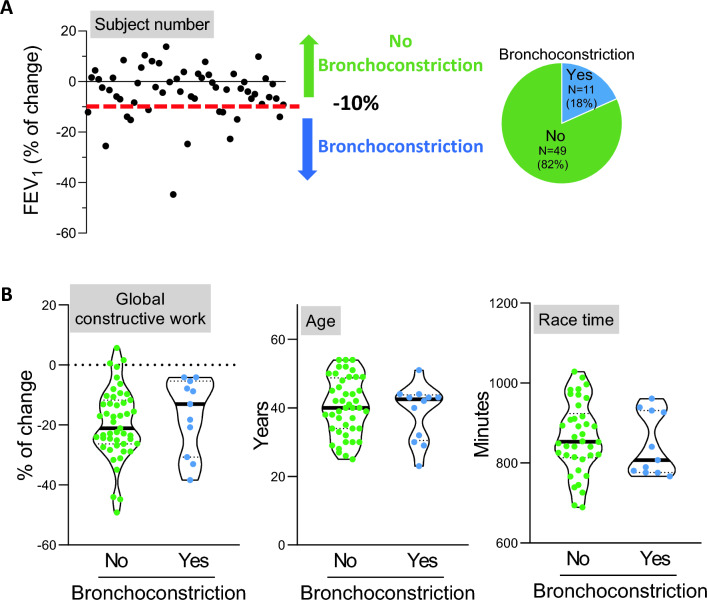
Exercise-induced bronchoconstriction. **A** Identification of triathletes with bronchoconstriction (defined as a post-race drop of > 10% in FEV_1_). **B** Violin representation of individual (points) and median (bold line) of percentage of change in global constructive work, age and race time in triathletes with or without bronchoconstriction. None of the parameters was significantly modified. *Significance *P* < 0.05 (unpaired *t*-test, adjusted on the basis of FDR) (*N* = 60). FEV_1_, forced expiratory volume in 1 s

The 11 athletes experiencing bronchoconstriction (Fig. 3A) displayed comparable post-race alterations in various left and right systolic cardiac function parameters, including left ventricle global work index and global constructive work (*P* = 0.481 and *P* = 0.514, respectively), compared with normal athletes (Fig. 3B, Table 3). Notably, they exhibited a significant 20% reduction in the left ventricle filling pressure index by *E*/*E*′ (*P* = 0.041). There were no discernible differences in age and race time between the two subgroups.

**Fig. 4:**
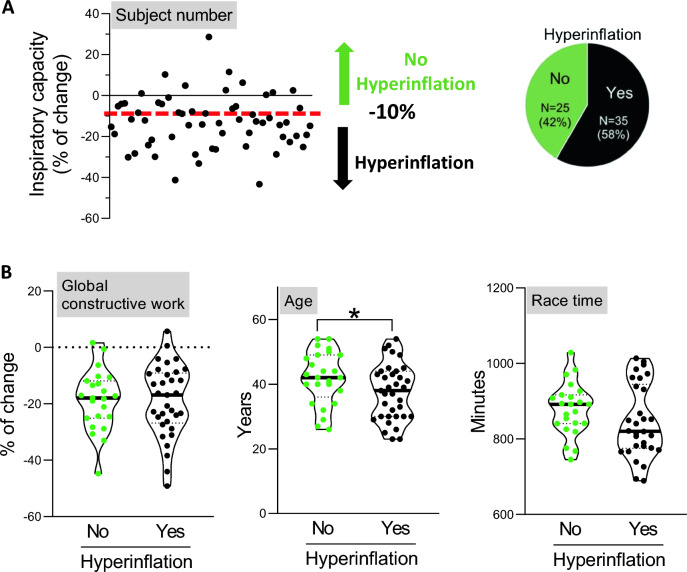
Exercise-induced dynamic hyperinflation. **A** Identification of triathletes with hyperinflation (defined as a post-race drop of > 10% in inspiratory capacity). **B** Violin representation of individual (points) and median (bold line) of percentage of change in global constructive work, age and race time in triathletes with or without hyperinflation. Population with bronchoconstriction was on average younger than the population without the bronchoconstriction. Global constructive work and race time were not different in this population. *Significance *P* < 0.05 (unpaired *t*-test, adjusted on the basis of FDR) (*N* = 60)

The 35 athletes exhibiting dynamic hyperinflation (Fig. 4A) also showed comparable changes in left and right systolic and diastolic cardiac function parameters. Global work index and global constructive work were also similar (*P* = 0.354 and *P* = 0.962, respectively) when compared with normal athletes (Fig. 4B, Table 3). On average, they were 5 years younger (*P* = 0.048) than athletes without hyperinflation but had similar race times.

**Fig. 5:**
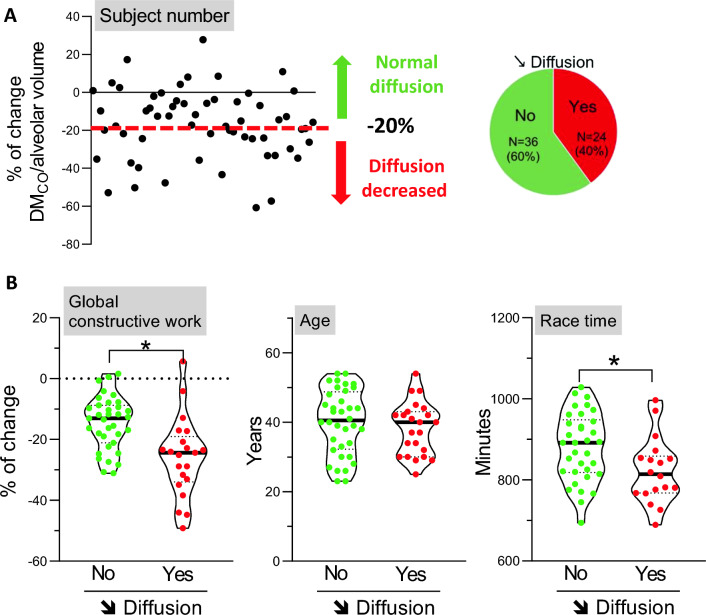
Alveolar–capillary membrane diffusion impairment. **A** Identification of triathletes with a marked decrease in DM_CO_/alveolar volume according to a − 20% cut-off. **B** Violin representation of individual (points) and median (bold line) of percentage of change in global constructive work, age and race time in triathletes with or without a marked decrease of membrane diffusion. Population with decreased alveolar–capillary membrane diffusion had on average reduced global constructive work and race time compared with the population without altered alveolar–capillary membrane diffusion. Age was not different in this population. *Significance *P* < 0.05 (unpaired *t*-test, adjusted on the basis of FDR) (*N* = 60). DM_CO_, alveolar–capillary membrane diffusing capacity for carbon monoxide

Twenty-four athletes exhibited impaired diffusing capacity, as indexed by a decrease in DM_CO_ per unit effective alveolar volume of > 20% (Fig. 5A). In comparison with their counterparts, these athletes demonstrated a more pronounced decline in global work index and global constructive work (*P* = 0.001 and *P* = 0.001, respectively) (Fig. 5B, Table 3). Notably, significant positive correlation was observed between the percentage change in diffusing capacity and global constructive work (Pearson *r* = 0.39, *P* = 0.004, Supplemental Fig. S1). The left ventricle filling pressure index by *E*/*E*′ increased by + 8% in athletes with impaired diffusing capacity, whereas it decreased by 10% in the other athletes (*P* = 0.025). Both subgroups were of the same age, but athletes with impaired diffusing capacity completed the race approximatively 1 h faster compared with those without changes in post-race diffusion (*P* = 0.021), mainly due to faster running time (*P* = 0.003). The percentage of change in DM_CO_/alveolar volume was negatively correlated with total race time (Pearson *r* = 0.33, *P* = 0.02, Supplemental Fig. S2).

## Discussion

This study was carried out to investigate associations between pulmonary and cardiac function changes following prolonged strenuous exercise. A reduction in the alveolar–capillary membrane diffusing capacity post-race was found to be associated with a decrease in left ventricle systolic function, as revealed by a more pronounced decrease in myocardial work in those athletes. However, changes in ventilation flows and volumes, including exercise-induced bronchoconstriction and dynamic hyperinflation post-race, were not associated with changes in cardiac function.

### Exercise-Induced Pulmonary Alterations and Cardiac Fatigue

We observed a reduction in both cardiac and pulmonary function after prolonged strenuous exercise. The exercise-induced cardiac fatigue was consistent with previous findings [27]. Furthermore, to evaluate left ventricle systolic function, we used new cardiac indexes of myocardial work which are less dependent on the haemodynamic and loading conditions, while providing prognostic information in the case of cardiac pathologies [28]. Our study revealed evidence of decreased myocardial work that was more marked than the decrease in left ventricle ejection fraction or global longitudinal strain, and increased global wasted work after the very long-distance triathlon race. There are currently very few data available regarding alterations in myocardial work after prolonged strenuous exercise. Erevik et al. reported similar results after a 91 km mountain bike leisure race, but without any change in left ventricle ejection fraction or global longitudinal strain [29]. They concluded that the myocardial work parameters were indicative of myocardial inefficiency that may precede a decrease in global left ventricle function, suggesting that myocardial work assessment could be a sensitive parameter to assess left ventricle function after long-duration races.

In our study, 18% of athletes demonstrated exercise-induced bronchoconstriction, while dynamic hyperinflation was observed in 58%. The delay in post-race evaluation seems to influence the number of athletes showing post-exercise bronchoconstriction. Zavorsky et al. reported a higher proportion of athletes with bronchoconstriction (29%) 25 min following a semi-marathon and marathon [30]. Therefore, we cannot determine if conducting echocardiography and spirometry sooner after the race would have revealed any connection to cardiac fatigue. Vernillo et al. documented a 21% reduction in inspiratory capacity favouring dynamic hyperinflation after a mountain ultramarathon [31]. This phenomenon could be attributed to progressive alveolar air trapping [9]. Our study also identified a significant alteration in alveolar–capillary oxygen diffusing capacity post-race, reflected in decreased diffusing capacity for CO and NO, consistent with findings of other studies [11]. The decline in diffusing capacity for CO and NO suggests impairment of the alveolo-capillary membrane conductance of gas. This is supported by reductions in DM_CO_ and DM_CO_ per unit effective alveolar volume, as well as a marginal decrease in *V*_cap_ per unit effective alveolar volume. While DM_CO_ and *V*_cap_ are computed variables, the decrease in the DL_NO_/DL_CO_ ratio post-race aligns with a reduction in the DM_CO_/V_cap_ ratio [32]. Unlike bronchoconstriction, diffusion impairment appears to be relatively stable for several hours after arrival and is less dependent on the timing of the examinations [33]. Although desaturation might be anticipated due to the reduction in membrane diffusing capacity post-race, our overall athlete population did not exhibit this phenomenon. This finding is consistent with previous reports demonstrating rapid pulsed arterial oxygen saturation recovery post-race [34]. Furthermore, the observed drop in DL_CO_ or DM_CO_ post-race was likely insufficient to impact pulsed arterial oxygen saturation [35].

### Relation Between Exercise-Induced Pulmonary and Cardiac Dysfunction

The comprehensive evaluation of both cardiac and pulmonary parameters in a substantial cohort was a notable strength of our study. A novel finding was the association between the post-race decrease in alveolar–capillary membrane conductance and the reduction in myocardial work. During exercise, increased cardiac output and pulmonary vascular pressure can elevate capillary permeability, potentially causing pulmonary oedema and capillary haemorrhage [36]. Post-exercise, pulmonary oedema can be gauged by the decline in alveolar–capillary membrane diffusing capacity, modification in X-ray lung density [10] or an increase in lung comet tails using transthoracic ultrasound [37]. Scant data exist on the relationships between pulmonary and cardiac function alterations following prolonged exercise. A lone study revealed that cardiovascular factors may contribute to pulmonary membrane conductance impairment post-exercise. Stickland et al. observed a positive correlation between the post-race drop in membrane diffusing capacity and post-race left ventricle systolic function evaluated in 12 endurance cyclists who underwent a 20 km simulated bicycle time trial [38]. Cyclists with the lowest left ventricle systolic function post-time trial displayed the most impaired pulmonary membrane conductance. In our study, the connection between altered membrane conductance and cardiac fatigue could potentially be mediated by increased left ventricular filling pressures and diastolic function alterations. Athletes experiencing the most significant impact on myocardial work also exhibited the greatest drop in left ventricle relaxation during the diastolic phase. Those with greater membrane conductance impairment post-race also demonstrated an increased E/E′ ratio. This impaired myocardial contractile function may be linked to elevated pulmonary capillary pressure, potentially leading to pulmonary interstitial oedema. Although the mechanism may be different, Kagami et al. recently demonstrated an increase in extravascular lung water during recovery from maximal exercise echocardiography in patients with heart failure with preserved left ventricle ejection fraction [39]. Interestingly, the membrane diffusion impairment was more likely observed in the fastest athletes. It could be anticipated that these abnormalities are related to higher race intensity and a greater cardiopulmonary stimulation. In the context of exercise-induced pulmonary oedema, exercise intensity seems to have a prominent role [40]. However, we lack information on the actual level of exertion intensity during the race, which would be possible, for example, by knowing the average percentage of heart rate during the race compared with the athlete’s maximum heart rate. Since the athletes run the marathon faster, it is unlikely that the observed membrane diffusion abnormality impacted the subject’s performance, and the present findings are therefore more in favour of a marker than a limitation of physical performance.

The absence of an association between bronchoconstriction and cardiac dysfunction following prolonged strenuous exercise in our study challenges the hypothesis of cardiac dysfunction influencing peribronchial congestion and bronchoconstriction, as documented in heart failure [16]. However, in the post-exercise context, no study findings have ever supported a potential link between exercise-induced bronchoconstriction and cardiac dysfunction. Conversely, dynamic hyperinflation may play a role in depressing left ventricle function in healthy subjects, particularly during the diastolic phase [41]. Air trapping increases end-expiratory pressure, exerting mechanical pressure on the heart and reducing venous return, as reported in chronic obstructive pulmonary disease [42]. Hyperinflation can also lead to pulmonary vascular compliance impairment, potentially reducing left ventricular pre-loading and, consequently, cardiac output [43]. Our study did not reveal any relationship between the inspiratory capacity drop and alterations in cardiac function. We propose that the hyperinflation phenotype observed after a race in healthy athletes may differ from that found in patients with chronic obstructive pulmonary disease (COPD), i.e. gas trapping in COPD versus decrease in expiratory time due to increased ventilatory requirements. In healthy subjects, voluntary hyperinflation during exercise has not been found to compromise cardiac function [44].

### Study Limitations

We acknowledge several limitations of our study. Some discrepancies between the Hyp'Air Compact PFT device used in our study and other devices such as the Jaeger MasterScreen Pro have been reported for DL_NO_ [45]. Considering that the same device has been used to compare pre- and post-race, the difference observed in the present cannot be explained by a device specificity. The values of DL_NO_ and DL_CO_ depend on the alveolar gas concentrations inhaled (before diffusion) and, therefore, could be affected by the inhomogeneities of the pulmonary ventilation/perfusion ratios that can occur in post exercise [46], but this assessment is not evaluable by field methods. Due to the observational nature of our study, we cannot establish causal relationships or precisely identify underlying mechanisms. While our data demonstrate links between cardiac and pulmonary dysfunction after an ultra-endurance triathlon, the exact mechanisms remain elusive. We propose an association between altered alveolar–capillary membrane diffusion capacity and increased myocardial workload mediated by elevated pulmonary capillary pressure. However, pulmonary capillary pressure was estimated using Doppler echocardiography alone. Future studies with right heart catheterization could confirm this hypothesis definitively. We defined hyperinflation as a decrease in inspiratory capacity, acknowledging that some authors have suggested that the decrease in inspiratory capacity may be more related to respiratory muscle fatigue than to hyperinflation itself [7]. This nuanced consideration warrants attention in interpreting our results. Differential effects depending on different modes of exercise (swimming, cycling, running, etc.) may affect differentially pulmonary function that was not been investigated in the present study and might be an area of further research. Lastly, our study included only men owing to limited participation of women in the race (30 of 1300 participants). Although the comparison between male and female athletes would be interesting, it has been shown that ultramarathons negatively impact respiratory function with larger effect sizes in males compared with similar performances [37]. Addressing these limitations in future investigations will contribute to a more comprehensive understanding of the observed associations and their implications.

## Conclusion

After prolonged strenuous exercise, triathletes exhibited a cardiac fatigue, as shown by a drop in myocardial work. At the pulmonary level, in some athletes we observed exercise-induced bronchoconstriction, dynamic hyperinflation and a decrease in alveolar–capillary membrane diffusing capacity. Whereas triathletes with or without bronchoconstriction or hyperinflation had similar cardiac function modification, our study highlights—in a large cohort—that the decrease in alveolar–capillary membrane diffusing capacity was associated with the reduction in myocardial work post-race. These data provide new insight into the relationships between pulmonary gas exchange abnormalities and cardiac fatigue after prolonged strenuous exercise. Although our results supported the hypothesis of subclinical pulmonary oedema, further studies including a precise assessment of pulmonary capillary pressure would be useful to enhance insight into the mechanisms underlying these complex relationships.

## Supplementary Information

Below is the link to the electronic supplementary material.

Supplementary file1 (DOCX 159 KB)

## Abbreviations

COPD: Chronic obstructive pulmonary disease
DL_CO_: Lung diffusing capacity for carbon monoxide
DL_NO_: Lung diffusing capacity for nitric oxide
DM: Alveolar–capillary membrane diffusing capacity
DM_CO_: Alveolar–capillary membrane diffusing capacity for carbon monoxide
FEV_1_: Forced expiratory volume in one second
SpO_2_: Pulsed arterial oxygen saturation
*V*_cap_: Pulmonary capillary blood volume

## Declarations

### Data Availability

The data that support the findings of this study are not openly available due to reasons of sensitivity and are available from the corresponding author upon reasonable request.
