# Supplementary material for: Cardiac Fatigue in Male Athletes with Exercise-Induced Pulmonary Impairments After a Very Long-Distance Triathlon
Source: Sports Med. 2024 Oct 16;55(3):739–51. doi: 10.1007/s40279-024-02128-8 (PMC11985568; doi:10.1007/s40279-024-02128-8)
Supplement: Supplementary file 1 — Supplementary file1 (DOCX 159 KB) [file 40279_2024_2128_MOESM1_ESM.docx]

**SUPPLEMENTARY INFORMATION**

**Cardiac fatigue in male athletes with exercise-induced pulmonary impairments after a very long-distance triathlon**

Christophe Hédon^a,b,c,*^, MD, Fares Gouzi^a,c^, MD, PhD, Caroline Padovani^b^, MD, Iris Schuster^a,d^, MD, PhD, Claire Maufrais^e^, PhD, Stéphane Cade^f^, MD, Frédéric Cransac^f^, MD, Gaspard Bui^c^, MD, Samuel Morcillo^c^, BSc, Bronia Ayoub^a,c^, MSc, Jérôme Thireau^a^, PhD, Omar Izem^e^, PhD, Cyril Reboul^e^, PhD, Guillaume Walther^e^, PhD, Maurice Hayot^a,c^, MD, PhD, Stéphane Nottin^e,#^, PhD, Olivier Cazorla^a,#^, PhD

**1. Supplemental Methods**

**1.2 Supplementary echocardiography methodology information**

Echocardiographic images were obtained using five commercially available ultrasound systems (Vivid IQ, Vivid S70 and Vivid E95, 3Sc-RS probe, General Electric). Images were obtained by Experienced sonographers followed a standardized protocol with participants in a left lateral decubitus resting position. Images, recorded with five ECG-triggered cardiac cycles, were analysed offline using EchoPac 203 software (General Electric), averaging data from three cardiac cycles. Left ventricle volume and ejection fraction were calculated using the biplane Simpson method. Stroke volume was determined from the aortic root area and integral of the aortic blood flow velocity. Doppler assessments, in the apical 4-chamber view, were used for left ventricle diastolic function: maximal velocity of mitral E and A waves (Peak E and Peak A), maximal velocity of E’ at lateral mitral annulus (Peak E’_lat_). Right ventricle dimensions and global systolic function were evaluated using fractional area change, tricuspid annular plane systolic excursion (TAPSE), and tricuspid annular systolic velocity from TDI (peak S’). Left ventricle global longitudinal strain was measured in apical 4-chamber, 2-chamber and 3-chamber views using semi-automatic tracking (AFI mode by General Electric) with exams rejected if tracking was insufficient in at least 2 segments. Left ventricle circumferential strain analysis were performed on short-axis views at basal and apex levels (Q-analysis mode by General Electric). Right ventricular free wall longitudinal strain was obtained in apical 4-chamber view (Q-analysis mode by General Electric).

Myocardial work and related indices were estimated using the AFI-mode in the vendor-specific module (EchoPac 203, General Electric). This involved integrating the left ventricle global longitudinal strain and intra-left ventricle pressure, estimated non-invasively from brachial systemic blood pressure measurements taken just before echocardiography, as described by Russel et al. [1]. A left ventricle pressure-strain loop curve was constructed, and additional parameters were calculated, including the global work index measured as the total work from mitral valve closure to opening, representing the pressure-strain loop area. Global constructive work was defined as myocardial work during segmental shortening in systole, and segmental lengthening during the isovolumetric relaxation phase. Global wasted work was the work performed during lengthening in systole and shortening in isovolumic relaxation associated with energy loss. Global work efficiency was expressed as the ratio between myocardial global constructive work and the sum of global constructive work and global wasted work.

**1.2 Supplementary pulmonary function testing methodology information**

Spirometry was conducted using a single spirometer (Medisoft-MGCd, Sorinnes, Belgium) in accordance with international recommendations [2]. For lung airway flows and volumes, a daily calibration of the spirometer using a 3 L syringe was performed before the first test and verified regularly during the day of the test. The ambient temperature, and barometric pressure were also checked, and all spirometry outcomes were reported at body temperature, ambient barometric pressure and saturated with water vapor (BTPS). In case of measurement drift, a new calibration was performed. A satisfactory end of forced expiration had to be recognized to take the result into account. A consistent operator performed slow vital capacity followed by forced expiration maneuvers, each repeated at least three times for each athlete. The measured spirometry parameters included forced expiratory volume in one second (FEV_1_), slow vital capacity, inspiratory capacity and inspiratory reserve volume. The test adhered to acceptability, usability, and repeatability criteria, with a double-blind assessment conducted by two investigators before and after the race for relevant measurements. The final analysis utilized the largest FEV_1_ and vital capacity observed from all validated values. Exercise-induced bronchoconstriction was defined as a post-race drop of >10% in FEV_1_ compared to the pre-race levels [3]. Dynamic hyperinflation of the lung was identified by a 10% decrease in the post-race inspiratory capacity, considered a clinically relevant cut-off [4].

The alveolar-capillary diffusing capacity was assessed as previously described [5], by studying the lung diffusing capacity for carbon monoxide (DL_CO_) and nitric oxide (DL_NO_) using the Hyp’Air system (Medisoft-MGCd, Sorinnes, Belgium). The spirometer and gas analyzers were calibrated each day according to the manufacturer’s requirements and verified regularly during the experiment. Subjects were in sitting position and wore a nose-clip. A 8L inspiratory bag was prepared before the measurement and gas concentrations were measured directly from this bag before the patient’s inhalation to total lung capacity. A mixture containing 0.28% CO, 13% He and 18% O_2_ balanced with N_2_ was mixed with a NO/N_2_ mixture. The final concentration of NO in the bag inspired was 50 ppm and that of O_2_ was 19.1%. Measurements for DL_CO_ and DL_NO_ were conducted simultaneously in duplicate, with a 4-min rest between each measurement, performed in duplicate, according to the ERS standardization for single-breath determination of nitric oxide uptake in the lung [6]. Athletes, after breathing normally, were instructed to exhale to the residual volume of their lungs, rapidly inspire the gas mixture to total lung capacity, hold their breath for at least 4 seconds, and then expire rapidly. After discarding the dead space (900 mL), the exhaled air was collected in a sampling bag (900 mL) for automatic analysis of NO, CO and He concentrations. The inspiratory volume had to be >85% of the vital capacity and the 4–6 s apnea time. Alveolar volume during breath-hold was calculated using the He-dilution technique. Total breath-holding time was determined according to the Jones and Meade formula [7], starting after the first 30% of inspiratory time and finishing halfway through the collection of the expired sample. In addition, DL_NO_ and DL_CO_ measurements were reported at BTPS and accepted if within 10% of each other. Given the lack of lung disease in the athletes, and assumption of normal hemoglobin concentration (14.6 g.dL^-1^ for males) and given the constant level of inspired oxygen at barometric pressure (685 mmHg and 688 mmHg at 871 m elevation on the test days), the alveolar-capillary oxygen partial pressure was estimated at 97 mmHg. The θ_NO_/θ_CO_ ratio (ψ) was set at 7.9.

The alveolar-capillary membrane diffusing capacity for carbon monoxide (DM_CO_) and pulmonary capillary blood volume (V_cap_) were calculated using the Roughton and Forster method [8] with the equation 1/DL_gas_ = 1/DM_gas_ + 1/(θ_gas_⋅V_cap_)], where θ_gas_ is the specific conductance in the blood for the gas. Thus,

- V_cap_ = [(1/θ_CO_)·(1-α/ψ)] / (1/DL_CO_- α/DL_NO_)
- DM_CO_ = (1/α-1/ψ) / (1/DL_NO_-1/(ψ·DL_CO_))

Following international standards [6], DM_CO_ was calculated as DM_NO_/α, where α =1.97. A finite value of θ_NO_ = 4,5mlCO/min/mmHg/ml_blood_ was employed to determine DM_CO_ and V_cap_ [9]. Guénard’s equation [10] was utilized to calculate θ_CO_, considering alveolar-oxygen pressure (PaO2) and haemoglobin level.

Athletes exhibiting a significantly altered alveolar-capillary membrane diffusing capacity were identified through a post-race decrease in DMCO per unit effective alveolar volume greater than 20%. This criterion was based on changes reported in symptomatic chronic heart failure patients [11, 12]. Pulsed arterial oxygen saturation (SpO2) was measured using a Masimo SET® Rad-5 pulse oximeter with a finger sensor (Masimo, Danderyd, Sweden).

We assessed the impact of pulmonary alterations on post-race cardiac function by differentiating subgroups of triathletes according to the post-race onset of: 1) exercise-induced bronchoconstriction, 2) dynamic hyperinflation or 3) marked DM impairment.

**Statistical analysis**

Quantitative variables are presented as median and interquartile range or mean ± standard deviation (SD). Qualitative variables are presented as numbers and percentages. Comparisons of cardiac and pulmonary parameter variations between the pre-race and post-race measurements were done using a paired Student’s t-test, after checking the normal data distribution. For all parameters, we assessed the percentage variation between the pre-race and post-race measurements as (post-race–pre-race)x100/ pre-race. Correlations between the percentage variation of cardiac and ventilatory function parameters were obtained using the Pearson correlation coefficient. Subgroup comparisons were done using unpaired Student's t-tests, after checking the normal data distribution, on the percentage of change in the cardiac and pulmonary parameters. All p values were adjusted based on the false discovery rate (FDR) for multiple tests applied to all p values together. Significance was set at 0.05 for all comparisons. The analyses were performed using GraphPad Prism software (version 8.2.1).

**REFERENCES**

1. Russell K, Eriksen M, Aaberge L, et al (2012) A novel clinical method for quantification of regional left ventricular pressure–strain loop area: a non-invasive index of myocardial work. Eur Heart J 33:724–733

2. Graham BL, Steenbruggen I, Miller MR, et al (2019) Standardization of Spirometry 2019 Update. An Official American Thoracic Society and European Respiratory Society Technical Statement. Am J Respir Crit Care Med 200:e70–e88

3. Parsons JP, Hallstrand TS, Mastronarde JG, et al (2013) An official American Thoracic Society clinical practice guideline: exercise-induced bronchoconstriction. Am J Respir Crit Care Med 187:1016–1027

4. O’donnell DE, Lam M, Webb KA (1998) Measurement of Symptoms, Lung Hyperinflation, and Endurance during Exercise in Chronic Obstructive Pulmonary Disease. Am J Respir Crit Care Med 158:1557–1565

5. De La Villeon G, Gavotto A, Ledong N, et al (2022) Double gas transfer factors (DLCO-DLNO) at rest in patients with congenital heart diseases correlates with their ventilatory response during maximal exercise. International Journal of Cardiology Congenital Heart Disease 8:100346

6. Zavorsky GS, Hsia CCW, Hughes JMB, Borland CDR, Guénard H, van der Lee I, Steenbruggen I, Naeije R, Cao J, Dinh-Xuan AT (2017) Standardisation and application of the single-breath determination of nitric oxide uptake in the lung. Eur Respir J 49:1600962

7. Jones RS, Meade F (1961) A Theoretical and Experimental Analysis of Anomalies in the Estimation of Pulmonary Diffusing Capacity by the Single Breath Method. Quarterly Journal of Experimental Physiology and Cognate Medical Sciences 46:131–143

8. Roughton FJW, Forster RE (1957) Relative Importance of Diffusion and Chemical Reaction Rates in Determining Rate of Exchange of Gases in the Human Lung, With Special Reference to True Diffusing Capacity of Pulmonary Membrane and Volume of Blood in the Lung Capillaries. Journal of Applied Physiology 11:290–302

9. Carlsen E, Comroe JH (1958) The rate of uptake of carbon monoxide and of nitric oxide by normal human erythrocytes and experimentally produced spherocytes. J Gen Physiol 42:83–107

10. Guénard HJ-P, Martinot J-B, Martin S, Maury B, Lalande S, Kays C (2016) In vivo estimates of NO and CO conductance for haemoglobin and for lung transfer in humans. Respiratory Physiology & Neurobiology 228:1–8

11. Magini A, Apostolo A, Salvioni E, Italiano G, Veglia F, Agostoni P (2015) Alveolar–capillary membrane diffusion measurement by nitric oxide inhalation in heart failure. European Journal of Preventive Cardiology 22:206–212

12. Agostoni P, Bussotti M, Cattadori G, Margutti E, Contini M, Muratori M, Marenzi G, Fiorentini C (2006) Gas diffusion and alveolar–capillary unit in chronic heart failure. European Heart Journal 27:2538–2543

**2. Supplemental results**

**Supplemental Table S1:** Prerace values in function of postrace exercise-induced bronchoconstriction, hyperinflation or diffusion impairment

|  | PRERACE VALUES IN FUNCTION OF EXERCISE-INDUCED | | | | | | | | |
| --- | --- | --- | --- | --- | --- | --- | --- | --- | --- |
|  | Bronchoconstriction | | Hyperinflation | | | | Diffusion impairment | | |
|  | no (n=49) | yes (n=11) | | no (n=25) | yes (n=35) | | | no (n=36) | yes (n=24) |
| General characteristics |  |  | |  |  |  | | |  |
| Age | 39±9 | 38±9 | | **42±8** | **37±9*** | 40±9 | | | 38±8 |
| BMI | 22.6±1.8 | 23.6±2.3 | | 22.8±2.2 | 22.8±1.8 | 22.9±2.1 | | | 22.6±1.8 |
| Years of triathlon experience | 7±5 | 10±8 | | 8±6 | 7±6 | 8±7 | | | 7±5 |
| N of ultratriathlon in the past | 1.2±1.6 | 1.5±2.3 | | 0.9±1.1 | 1.5±2.0 | 1.2±1.6 | | | 1.1±1.6 |
| Pulmonary function |  |  | |  |  |  | | |  |
| SpO_2_ | 97±1 | 97±1 | | 97±1 | 97±1 | 97±1 | | | 97±1 |
| FEV_1_ (L) | 4.6±0.5 | 4.5±0.5 | | 4.5±0.7 | 4.6±0.5 | 4.5±0.5 | | | 4.7±0.5 |
| Inspiratory capacity | 2.5±0.6 | 2.5±0.6 | | 2.4±0.5 | 2.6±0.6 | 2.5±0.5 | | | 2.7±0.6 |
| DM_CO_/alveolar volume | 37±11 | 33±8 | | 35±11 | 37±10 | 36±6 | | | 39±11 |
| LV function |  |  | |  |  |  | | |  |
| LV end-diastolic volume (mL) | -157±22 | 172±34 | | -153±25 | 165±24 | 156±24 | | | 167±25 |
| LV ejection fraction (%) | 68±5 | 67±5 | | 69±4 | 67±5 | 67±4 | | | 69±5 |
| Cardiac output (l.min^-1^) | 6.0±1.5 | 5.8±0.9 | | 5.7±1.5 | 6.0±1.2 | 5.9±1.2 | | | 5.9±1.5 |
| E/E’_lat_ | 6.3±1.7 | 6.6±1.4 | | 6.4±2.0 | 6.1±1.4 | 6.6±1.8 | | | 5.7±1.2 |
| LV longitudinal strain (%) | -21.3±1.9 | -20.9±1.8 | | -21.1±1.8 | -21.5±2.0 | -21.0±1.9 | | | -21.7±1.9 |
| LV basal circumferential strain (%) | -18.3±3.3 | -19.7±1.7 | | -18.0±2.8 | -18.3±3.8 | -17.8±3.8 | | | -18.8±2.6 |
| LV apical circumferential strain (%) | -23.2±3.0 | -22.2±3.2 | | -23.1±4.3 | -22.9±3.6 | -22.9±3.6 | | | -23.1±4.3 |
| Global work index (mmHg.%) | **1993±200** | **2184±217*** | | 1996±195 | 2065±220 | 2022±220 | | | 2061±197 |
| Global constructive work (mmHg.%) | 2269±217 | 2410±210 | | 2279±224 | 2319±230 | 2297±248 | | | 2312±190 |
| RV function |  |  | |  |  |  | | |  |
| RV end-diastolic area (cm^2^) | 26±5 | 28±5 | | 25±5 | 27±5 | 26±4 | | | 26±5 |
| TAPSE (mm) | 28±5 | 27±3 | | 27±4 | 28±5 | 28±5 | | | 27±4 |
| RV peak S’ (cm.s^-1^) | 12±2 | 11±2 | | 12±2 | 12±2 | 12±2 | | | 12±1 |
| RV longitudinal strain (%) | -26.8±4.3 | -26.6±3.7 | | -26.0±4.4 | -27.3±3.6 | -26.3±4.4 | | | -27.4±3.2 |

Subgroups were clustered according to the presence or not of exercise-induce bronchoconstriction, dynamic hyperinflation and alveolar-capillary membrane diffusing capacity impairment after the race.

Values are presented as mean±SD.

BMI; body mass index; DM_CO_, alveolar-capillary membrane diffusing capacity for carbon monoxide; FEV_1,_ forced expiratory volume in one second; LV, left ventricular; RV, right ventricular; SpO_2_, pulsed arterial oxygen saturation; TAPSE, tricuspid annular plane systolic excursion.

* and bold means statistical difference p<0.05 assessed by unpaired t-test, adjusted based on FDR between the conditions yes and no (n=60).

**Supplemental Fig. S1** Correlation between the percentages of change of DM_CO_/alveolar volume and global constructive work, in the overall triathlete population (Pearson correlation test)

**Supplemental Fig. S2** Correlation between the percentage of change in DM_CO_/alveolar volume and total race time, in the overall triathlete population (Pearson correlation test)
